# Comprehensive analysis of the MYB transcription factor gene family in *Morus alba*

**DOI:** 10.1186/s12870-022-03626-5

**Published:** 2022-06-08

**Authors:** Li Liu, Nan Chao, Keermula Yidilisi, Xiaoru Kang, Xu Cao

**Affiliations:** 1 Jiangsu Key Laboratory of Sericultural Biology and Biotechnology, School of Biotechnology, Jiangsu University of Science and Technology, Zhenjiang, 212018 Jiangsu China; 2 Key Laboratory of Silkworm and Mulberry Genetic Improvement, Ministry of Agriculture and Rural Affairs, Sericultural Research Institute, Chinese Academy of Agricultural Sciences, Zhenjiang, 212018 Jiangsu China

**Keywords:** Anthocyanin, Fruit ripening, Functional annotation, *Morus alba*, MYB, Phenylpropanoid

## Abstract

**Background:**

The V-myb myeloblastosis viral oncogene homolog (MYB) family of proteins is large, containing functionally diverse transcription factors. However, MYBs in *Morus* are still poorly annotated and a comprehensive functional analysis of these transcription factors is lacking.

**Results:**

In the present study, a genome-wide identification of MYBs in *Morus alba* was performed*.* In total 166 *MaMYB*s were identified, including 103 *R2R3-MYB*s and four *3R-MaMYB*s. Comprehensive analyses, including the phylogenetic analysis with putative functional annotation, motif and structure analysis, gene structure organization, promoter analysis, chromosomal localization, and syntenic relationships of *R2R3-MaMYB*s and *3R-MaMYB*s, provided primary characterization for these *MaMYB*s. *R2R3-MaMYB*s covered the subgroups reported for *R2R3-MYB*s in *Arabidopsis* and *Populus,* and had two *Morus*-specific subgroups, indicating the high retention of MYBs in *Morus*. Motif analysis revealed high conservative residues at the start and end of each helix and residues consisting of the third helix in R2 and R3 repeats. Thirteen intron/exon patterns (a–m) were summarized, and the intron/exon pattern of two introns with phase numbers of 0 and 2 was the prevalent pattern for *R2R3-MaMYB*s. Various cis-elements in promoter regions were identified, and were mainly related to light response, development, phytohormone response, and abiotic and biotic stress response and secondary metabolite production. Expression patterns of *R2R3-MaMYB*s in different organs showed that *MaMYBs* involved in secondary cell wall components and stress responsiveness were preferentially expressed in roots or stems*. R2R3-MaMYB*s involved in flavonoid biosynthesis and anthocyanin accumulation were identified and characterized based on functional annotation and correlation of their expression levels with anthocyanin contents.

**Conclusion:**

Based on a comprehensive analysis, this work provided functional annotation for R2R3-MYBs and an informative reference for further functional dissection of MYBs in *Morus*.

**Supplementary Information:**

The online version contains supplementary material available at 10.1186/s12870-022-03626-5.

## Background

V-myb myeloblastosis viral oncogene homolog (MYB) proteins comprise one of the largest families of transcriptional regulators in plants [1]. The MYB transcription factors have been identified as important regulators that work as activators or repressors in diverse processes, including development, stress responses, and metabolism. The “oncogene” v-Myb derived from the avian myeloblastosis virus was the first *MYB* gene identified and further study showed that three genes related to v-Myb-c-Myb, A-Myb and B-Myb were distributed in many vertebrates [2]. Similar genes have also been widely reported in fungi, plants, and insects [3]. The first plant MYB transcription factor the *c1* gene was reported in maize (*Zea mays*) [4]. Currently, many MYB genes have been reported in diverse species [5].

MYB proteins are characterized by sharing a highly conserved DNA-binding domain (DBD) in their N-terminus This domain is generally composed of up to four imperfect repeats [1, 6]. These repeat units are named R1, R2, and R3 according to their sequence similarity with the c-MYB protein [6]. Each repeat comprises approximately 50–53 amino acids with a similar folding architecture containing three well-defined α-helices. When bound to specific promoter sequences, portions of the second and third helices form a helix-turn-helix (HTH) structure [6, 7]; The third helices of R2 and R3 play a recognition role in binding to a specific DNA sequence, while R1 is not essential for specific DNA recognition [6, 7]. In addition, each repeat contains regularly spaced tryptophan residues (WX_18–19_WX_18–19_W), forming a tryptophan cluster in the three-dimensional (3D) HTH structure. The C terminus, which is responsible for the protein’s regulatory activity, is more variable [1, 8]. MYB transcription factors have been divided into four different classes depending on the number of adjacent repeats: 1R (R1/2, R3-MYB), 2R (R2R3-MYB), 3R (R1R2R3-MYB), and 4R, which harbor four R1/R2-like repeats. MYBs with two repeats R2R3-MYBs which is the largest class of MYB factors in plants [9].

*R2R3-MYB*s have undergone extensive expansion and are thought to perform diverse functions in plant-specific processes [5]. Comprehensive analysis of the *MYB* gene family have been widely reported in many plants that have available genome information. To date, 126 *2R-MYB* genes are reported in *Arabidopsis thaliana* [1], 198 in *Populus trichocarpa* [10], 108 in *Vitis vinifera* [11], 157 in *Z. mays* [12], and 185 in *Pyrus bretschneideri* [13]. R2R3-MYBs have been classified into 28 (S1–S28) subgroups in *Arabidopsis* and 45 subgroups (C1–C45) in *Populus trichocarpa* [1, 10]. Most R2R3-MYBs in *Arabidopsis* have been well functionally characterized and their roles in diverse biological processes have been identified. The functions of R2R3-MYBs in the regulation of the phenylpropanoid, flavonoid, and lignin biosynthesis pathways are particularly well studied [9, 14]. The first plant MYB transcription factor, the *c1* was found to regulate anthocyanin biosynthesis in the aleurone of maize kernels [4]. *Arabidopsis* R2R3-MYBs in subgroups 6 and 7 were also reported to control the flavonoid biosynthesis pathway, with differences in tissue preference [1]. AtMYB75/ PAP1, AtMYB90/PAP2, AtMYB113 and AtMYB114 in subgroup 6 control anthocyanin biosynthesis in vegetative tissues and AtMYB11/PFG1, AtMYB12/PFG1 and AtMYB111/PFG3 in subgroup 7 control flavonol biosynthesis in all tissues [15]. AtMYB123/TT2 in subgroup 2 controls the biosynthesis of proanthocyanidins (PAs) and regulates tannin biosynthesis [1]. In addition, some *R2R3-MYB*s also act as repressors for controlling the lignin and flavonoids biosynthesis pathway. AtMYB3 and 4 in subgroup 4 suppress the cinnamate 4-hydroxylase (*C4H*) gene, and AtMYB4 represses the synthesis of sinapoyl malate [14]. *AtMYB32* represses lignin and flavonoid biosynthesis in pollen. *AtMYB7* is responsible for reducing flavonol by regulating flavonol synthase (FLS) [14]**.** *PtMYB165*, *181*, *182,* and *194* in C18 were also reported as repressors of flavonoid biosynthesis [14]. In addition, *R2R3-MYB*s also serve as key factors in the control of plant development and cell fate, as well as various abiotic stresses and defense responses in plants [1]. *AtMYB0* and *AtMYBB66* are involved in epidermal cell patterning and *AtMYB125* is involved in the regulation of male gametophyte development by controlling the cell cycle [16, 17]. *AtMYB1*, *AtMYB2*, *AtMYB30*, *AtMYB41*, and AtMYB44 were reported to respond to different stresses [18–21].

Mulberry (*Morus*, Moraceae) is an economically important plant with nutritional, medicinal, and ecological value. A draft genome of *Morus notabilis* was released by He et al. (2013) [22], after which genome-wide analyses of several gene families, including the *R2R3-MYB* gene family, were performed [23]. The functions of four *R2R3-MYB*s in flavonoid biosynthesis were also characterized [24]. However, MYBs in *Morus* are still poorly annotated. In the present study, we performed a comprehensive analysis of *R2R3* and *3R-MYB*s using the latest chromosome-level genome assembly of *Morus alba* (Ma), which has a basic chromosome number of 14 and total genome assembly size of 338.27 Mb [25].. A strict workflow was adopted to perform a genome-wide identification of *MYB*s in *M. alba*. Comprehensive analyses, including phylogenetic analysis, putative functional annotation, gene structure, motif and promoter analysis, chromosomal localization and the syntenic relationships of *R2R3* and *3R*-*MaMYB*s, were performed. The expression patterns of *R2R3* and *3R*-*MaMYB*s in different organs and fruit development stages were analyzed using RNA-seq data. Real-time quantitative PCR (RT-qPCR) was used to validate selected differentially expressed *R2R3-MYB*s in different fruit development stages. This study contributed basic functional annotation of *R2R3*-*MaMYB*s in *Morus* and provided candidate *MYB* regulators as targets for genetic modification.

## Materials and methods

### Plant materials

Roots, stems and leaves were collected from one-year-old *M. alba* plants. Fruits at four different development stages (S0, inflorescence; S1, green fruits; S2, reddish fruits; S3, purple fruits) were collected from Zhongsang 5801 plants. All of the above samples were collected from the plantation of National Mulberry Genebank (NMGB), Zhenjiang, China, and immediately stored at − 80 °C. Three biological replicates growing nearby were considered. At least six mulberry fruits were collected from each plant.

### Data collection and workflow for searching MYBs in *M. alba*

Genome sequences were collected from different databases. The *Arabidopsis* genome sequences (.fasta) and annotation file (.gff) were downloaded from The Arabidopsis Information Resource (TAIR, https://www.arabidopsis.org/), and the *Populus* genome information was obtained from Phytozome v13 (https://phytozome-next.jgi.doe.gov/). The *M. alba* genome sequences (.fasta) and annotation file (.gff) were generously provided by Professor Jiao, who released this genome information. The MYBs in *Arabidopsis* and *Populus* were extracted using Tbtools v1.098665 [26] based on the information provided in previous reports [1, 8, 10].

To search for MYBs in mulberry, firstly, HMMER search was performed using a hidden Markov model (HMM) profile of the MYB binding domain (PF00249) from the Pfam database (http://pfam.xfam.org/). The hits with E values < 1 were screened, and the short open reading frames (length < 100) were filtered out and confirmed with manual speculation. The filtered sequences were screened as primary candidate MYBs. Secondly, the putative protein sequences of primary candidate MYBs were submitted to a simple MEME wrapper assembled in Tbtools v1.098665 using the following parameters: number of motifs to find 3, minimum motif width 6, and maximum motif width 55. MEME motifs were extracted and the MYB DNA binding domains were further validated manually. The primary candidate MYBs with MYB DNA binding domain were selected as MYBs in mulberry.

### Sequence alignment, motif analysis and homologous modeling

*Morus alba* (Ma) R2R3-MYBs and 3R-MYBs were aligned using clustal W assembled in MEGA7.0. The alignment result was exported and manually speculated for scanning the R1, R2 and R3 repeats. Seqlogos of this domain were generated using TBtools v1.098665. Protein sequence of DBD domain of R2R3-MYB, MaMYB29, was submitted to SWISS-MODEL (https://swissmodel.expasy.org/) to obtain the 3-D structure model using AtMYB66 (WER, PDB accession number: 6kks) as template. The models were submitted to SAVES (http://services.mbi.ucla.edu/SAVES/) and Chiron (http://troll.med.unc.edu/chiron/login.php) for further evaluation and modification. In addition, PSVS (https://montelionelab.chem.rpi.edu/PSVS/) was also adopted to further evaluate the quality of modeled structure and calculate the Ramachandran plot. Pymol was used to visualize the 3-D structure [27].

### Gene structure and promoter analysis of *R2R3* and *3R-MYB*s

The gene structure of each *MaMYB* was displayed based on the genome sequence and its annotation file using Gene Structure View assembled in Tbtools v1.098665. The upstream 2000 bp sequences were extracted for in silico promoter region analysis. Cis-acting elements were predicted using PlantCARE (http://bioinformatics.psb.ugent.be/webtools/plantcare/html/) (Lescot et al., 2002).

### Phylogenetic analysis and functional annotation of *R2R3* and *3R-MYB*s

A neighbor-joining (NJ) phylogenetic tree was constructed using full-length R2R3-MYB protein sequences from *A. thaliana*, *Populus* and *Morus* using MEGA7.0 [28] with JTT + G model and bootstrap test with 1000 replicates. The other NJ tree was also constructed using only R2R3-MaMYB proteins and 3R-MaMYBs with the same parameters.

### Chromosomal location and synteny analysis of *R2R3* and *3R-MYB*s

Chromosome location information of *R2R3* and *3R-MYB*s was extracted from *Morus alba* genome annotation file. BLASTP and Multiple Collinearity Scan toolkit (MCScanX) assembled in Tbtools v1.098665 were used to identify syntenic blocks, tandem duplication, and distinct duplication events using default parameters [29, 30]. *R2R3* and *3R-MaMYB*s were mapped to *Morus* chromosomes and displayed using Tbtools v1.098665 and both the tandem duplication and block duplication gene pairs were marked.

### Transcriptome based expression profile analysis

Our previous reported RAN-seq data (Accession number: PRJNA660559) was used to obtain the expression profiles of *R2R3* and *3R*-*MaMYB*s in roots, stems and leaves [31]. Expression profile of *R2R3* and *3R-MaMYB*s in different fruit development stages was obtained using RNA-seq data with accession number: CRA006074 in national genomics data center (NGDC). Both above RNA-seq datasets were reanalyzed. Chromosome-level *M.alba* genome was used as reference genome for alignment using bowtie2 [32]. The genome annotation file was used for calculating the expression matrix using StringTie v2.15 [33]. Differential expressed genes (DEGs) were obtained using DEseq2 by comparing expression levels of each two stages [34]. Organ expression preference genes were identified while ABS (log_2_EXPi - log_2_EXPj) > =1 (i, j indicating different organs) were obtained.

### RNA extraction and RT-qPCR analysis

RNA extraction and cDNA synthesis were performed as our previous report using Plant RN52 Kit (Aidlab, Beijing, China) and PC54-TRUEscript RT kit (Aidlab, Beijing, China) according to the manual [35]. qRT-PCR (quantitative real-time PCR) was performed to validate the expression patterns of *R2R3-MaMYB*s in different fruit development stages using ABI StepOnePlus™ Real-Time PCR System (USA). The primers are available in Additional file 1. *Actin* was used as reference gene [36]. Graphpad Prism8.0 was used to visualize the qRT-PCR results. SPSS19.0 was used to perform T-test and ANOVA, *p* < 0.05 was marked as significance. Three biological replicates were firstly mixed and three technical replicates respectively were performed for qRT-PCR.

### Measurement of anthocyanins content in mulberry fruits

Extraction and measurement of anthocyanins content were performed according to our previous study [35]. The anthocyanins content was given in cyanidin-3-glucoside equivalents.

## Results

### Identification of MYBs in *M. alba*

Based on our workflow, 166 genes encoding MaMYBs were identified in *M. alba*. Among 166 *MYB* genes, there were 59 *1R-MYB*s (named *MaMYBR1–59*), 103 *R2R3-MYB*s (named *MaMYB1–103*) and 4 *3R-MYB*s (named *MaMYB3R1–4*); and *4R*-*MYB* was not found in *M. alba* (Table 1 and Additional file 2). Some *MYB* genes had alternative splicing and generated more than one transcript. The alternative splicing occurred more frequently for *1R-MYB*s in *M. alba*. Similar alternative splicing events were also reported in *Arabidopsis* and *Populus MYB*s. A previous study identified 124 MYB-related genes, 116 *R2R3-MYB*s, and 4 *3R-MYB*s using a de novo transcriptome of *M. alba* [23]*.* This study used a chromosome-level genome and provided stricter and accurate recognition of MYBs in *M. alba*. Both R2R3-MYBs and 3R-MYBs contained DNA-specific recognition helices, and R2R3-MYBs were the largest class of MYBs in plants, playing diverse roles in plant specific processes. Therefore, we mainly focused on the investigation of 103 R2R3-MYB and 4 3R-MYB family members in this study.

**Table 1:** *MYB*s in different plants

Types	*Arabidopsis*	*Populus*	*Vitis*	*Morus*
1R	64	152	46	59(88)
2R(R2R3)	126	196	108	103(108)
3R	5	5	5	4(7)
4R	1	1	1	0
Ref	[1]	[10]	[11, 37]	This study

The number in brackets indicates the number of MYB transcripts in *Morus alba*

### Characterization of R2R3 and 3R-MaMYBs in *M. alba*

**Fig. 1:**
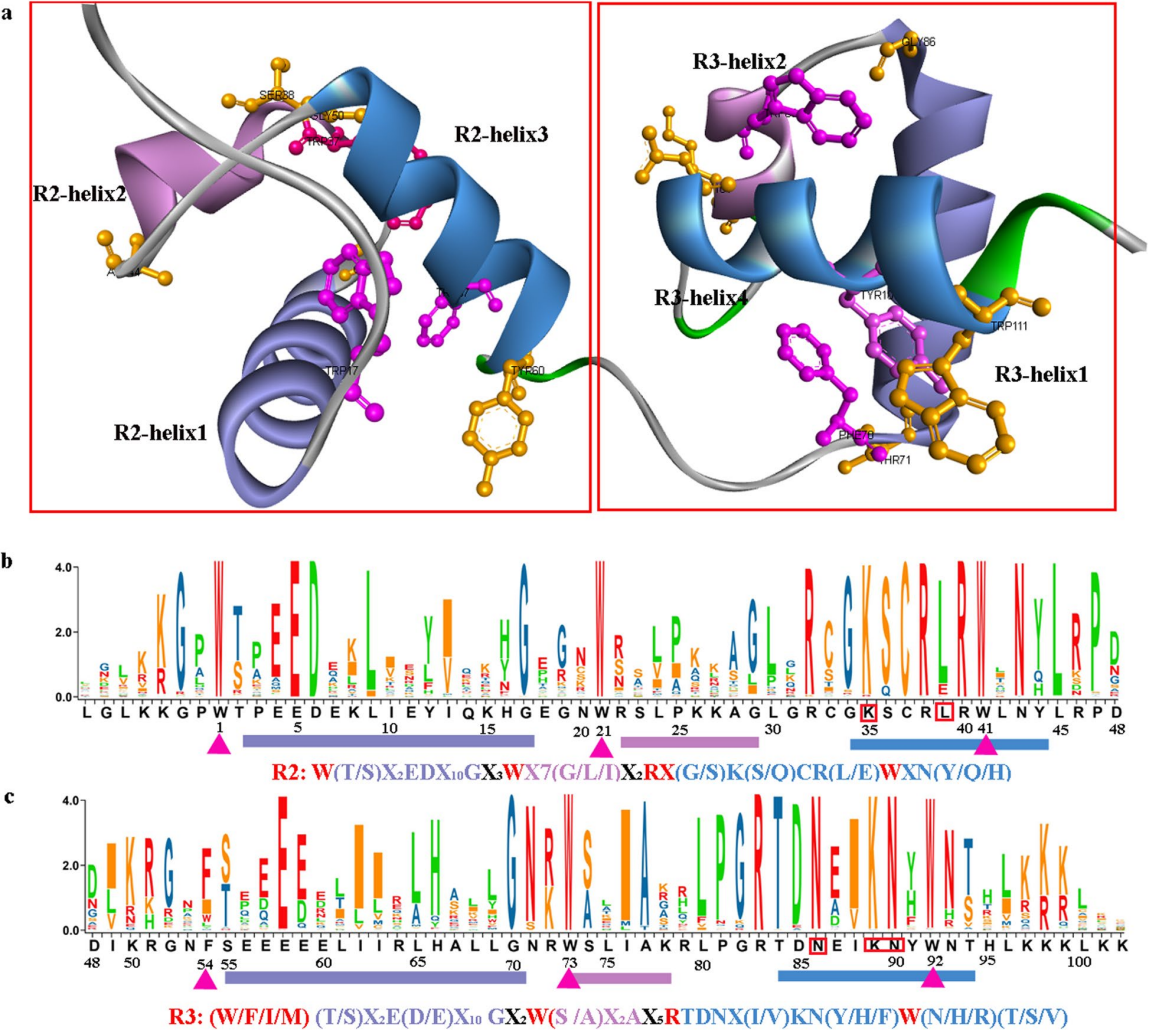
Motif analysis and structural characterization of R2R3-MaMYBs. **A** The three-dimensional structure of MaMYB29. Helices in R2 and R3 are indicated, and residues at the start and the end of each helix are shown as sticks and balls. **B** Sequence logos of the R2 repeat of R2R3-MaMYBs. **C** Sequence logos of the R3 repeat of R2R3-MaMYBs. Helices 1, 2, and 3 are indicated with colored lines below in purple, red, and blue, respectively, and the modified patterns for R2 and R3 are also indicated. Conserved W and F/I/M are indicated by solid purple triangles

Alignment was performed using 115 R2R3 and 3R-MYBs, including the alternative splicing products. The DBD domain was recognized and analyzed based on the alignment result. It was evident that all of these MaMYBs contained DBD domains and two or three repeats could be identified easily (Additional file 2). The DBD 3D structure of MaMYB29 was constructed using AtMYB66 (PDB accession number: 6kks) as a template to identify the helix regions (Fig. 1A). Helices 1, 2, and 3 are shown in Fig. 1. The highly conserved spaced W residues formed the WX_19_WX_19_W motif in R2 repeats (Fig. 1B, Additional file 2). Several R2R3-MYBs showed minor differences in the number of spaced amino acids. MaMYB5, 32, 63, 64, 70, and100 had additional G, forming WX_18_GXWX_19_W, and MaMYB13 had additional P and L residues, forming WX_19_WX_9_PLX_10_W. The additional amino acids were beyond the helix structures according to the helix regions identified (Fig. 1 and Additional file 2). The spaced W residues in R3 repeats were variable especially at the first W (site 54 in Fig. 1C), although W is still dominant amino acid. The (W/F/I/ M)X_18_WX_18_W was recognized in MaMYB R3 repeats (Fig. 1C). The replacement of the first W with F, (less frequently) I, or M in R3 was also reported in *Populus*, *Z. may, Arabidopsis,* and *Vitis* [1, 10–12]. MaMYB35 and 37 had replacement of the third W with Y and F, respectively. More conservative amino acids were observed in the third helix region of R2 and R3 repeats than those consisting of the first and second helices (Fig. 1B, C). In addition to the conserved W, there were also several highly conserved amino acids including the amino acids at the start and the end of each helix and amino acids consisting of the third helix in R2 and R3 repeats. In addition, the important MYB function related residues K35, L39, N86, K89, and 90, indicated by red boxes, were also highly conserved. Therefore, we could further modify the three helix regions to W(T/S)X_2_EDX_10_GX_3_WX_7_(G/L/I)X_2_RX (G/S)K(S/Q)CR(L/E) WXN(Y/Q/H) for R2 and (W/F/I/M)(T/S)X_2_E(D/E)X_10_ GX_2_W(S/A)X_2_AX_5_RTDNX(I/V)KN(Y/H/F)W(N/ H/R)(T/S/V) for R3 (Fig. 1B, C). Interestingly, it was clear that the patterns, W(T/S)X_2_EDX_10_G for R2 and (W/F /I/M)(T/S)X_2_E(D/E)X_10_G for R3 that formed the first helix were almost same for both R2 and R3 repeats. These patterns were annotated as InterPro MYB domain signature patterns (PS00037). In contrast, the patterns, (G/S)K(S/Q)CRWXN(Y/Q/H) for R2 and TDNX(I/V)KN(Y/H/F)W(N/H/R) (T/S/V) for R3, formed the third helices and were quite different for R2 and R3 repeats. The above results indicated that the conservation of the third helix in R2 or R3 preserved the DNA binding function and the difference in the third helix between R2 and R3 guaranteed recognition for specific DNA sequences. The WX_19_W_18_W box was detected for MaMYB3Rs and formed the R1 repeat.

### Phylogenetic analysis and functional classification of R2R3 and 3R-MaMYBs

Phylogenetic analysis of R2R3 and 3R-MYBs in *Arabidopsis*, *Populus,* and *Morus* showed that MaMYBs clustered well with orthologs in *Arabidopsis* or *Populus*. Previous studies have divided R2R3-AtMYBs into 28 subgroups (S1–S28) and R2R3-PtrMYBs into 45 subgroups (C1–C48). R2R3-MaMYBs covered all 28 R2R3-AtMYB subgroups and 44 R2R3-PtrMYB subgroups, except for C13 (Table 2, Additional file 3). In addition, MaMYB65 and 101 in addition to MaMYB53 and 54 (TT2L3) were classified as two *Morus* specific subgroups. MaMYB54, previously named as TT2L3, was reported to show a similar expression pattern with flavan-3-ol specific gene *LAR* encoding leucoanthocyanidin reductase during fruit ripening in HG2 [24]. MaMYB59, 73, and 76 belonging to C17 (S4) and MaMYB39 (MYB4) belonging to C18 were annotated as repressors of the phenylpropanoid pathway. AtMYB3, 4, and 32 in S4 were reported to be involved in suppressing lignin or flavonoid biosynthesis [14]. PtrMYB165, 182, and 194 in C18 were characterized as repressors for anthocyanin and proanthocyanidin biosynthesis [14]. MaMYB39, previously named MYB4, can negatively affect both anthocyanin and proanthocyanidin accumulation [24]. MaMYB42, 61, and 75 belonging to C19 (S7), MaMYB83 belonging to C12, MaMYB50, 81, 82, and 99 belonging to C25 (S5), and MaMYB58 belonging to C27 (S6) are annotated as flavonoid related genes based on the functions of their orthologs in *Arabidopsis* and *Populus* (Table 2). Other R2R3-MaMYBs with different annotated functions such as responsiveness to abiotic and biotic stresses are also displayed in Table 2.

**Table 2:** Classification of MaMYBs based on phylogenetic analysis

Subgroups	***Arabidopsis***	***Populus***	***Morus***	Function annotation	Reference
C1(S11)	AtMYB41, 49, 74 and 102	PtrMYB017, 043, 047, 226	MaMYB14 and 48	drought response and suberin biosynthesis	[38–40]
C2(S9)	AtMYB16, 106	PtrMYB038, 138 and 186	MaMYB11, 38 and 94	cuticular wax biosynthesis	[41]
C3(S10)	AtMYB9, 39 and 107	PtrMYB059, 130 and 149	MaMYB19	suberin biosynthesis and transport	[42]
C4(S24)	AtMYB53, 92 and 93	PtrMYB027, 030, 032 and 109	MaMYB28, 34 and 60	suberin biosynthesis	[43]
C5	AtMYB40	PtrMYB139 and 188	MaMYB6	arsenic resistance	[44]
C6	AtMYB20 and 43	PtrMYB018 and 152	MaMYB18	secondary cell wall formation and lignification	[45–47]
C7	AtMYB42 and 85	PtrMYB075, 92, 125, 199	MaMYB22 and 31	cell wall production	[48]
C8(S9)	AtMYB17	PtrMYB077, 146, 187and 209	MaMYB20 and 97	meristem identity transition from vegetative growth to flowering	[49]
C9(S1)	AtMYB30, 31 and 60	PtrMYB053, 81, 155 and 225	MaMYB10, 44 and 90	cell death during the hypersensitive response upon pathogen attack	[50, 51]
C10(S2)	AtMYB13, 14 and 15	PtrMYB185, 190 and 220	MaMYB43 and 77	cold or wound stress	[52, 53]
C11(S3)	AtMYB10, 58, 63 and 72	PtrMYB028 and 129	MaMYB86	lignin biosynthesis	[54]
C12	NA	PtrMYB048, 049, 063, 064 and 065	MaMYB15 and 16	–	–
C13	NA	PtrMYB016, 44, 45, 46 and 230	NA	–	–
C14	AtMYB35	NA	MaMYB9	sex determination	[55, 56]
C15	AtMYB8	PtrMYB005 and 094	MaMYB1	–	–
C16(S14)	AtMYB36, 37, 38, 68, 84 and 87	PtrMYB025, 079, 085, 088, 099100, 102,112, 133, 184	MaMYB5, 32,46,63, 64, 70 and 100	transition from proliferation to differentiation	[57, 58]
C17(S4)	AtMYB3, 4, 6, 7, 8 and 32	PtrMYB057, 093, 156, 168, 180 and 221	MaMYB59, 73 and 76	General phenylpropanoid and lignin R2R3-MYB repressors	[14]
C18		PtrMYB165, 181, 182, 194 and 203	MaMYB39(MYB4)	Flavonoid R2R3-MYB repressors	[14]
C19(S7)	AtMYB11, 12 and 111	PtrMYB035, 056 and 111	MaMYB42, 61 and 75(MYBF)	flavonoids biosynthesis	[9, 24]
C20	AtMYB5	PtrMYB006, 050, 060, 061, 107 and 126	MaMYB83	flavonoids biosynthesis	[9]
C21	NA	PtrMYB009, 115, 123, 153 and 201	MaMYB98	proanthocyanidin biosynthesis and enhances fungal resistance	[9]
C22	AtMYB82	PtrMYB084 and 135	MaMYB4	response to low oxygen, trichome development	[59]
C23(S15)	AtMYB0, 23 and 66	PtrMYB071, 072 and 143	MaMYB36 and 89	anthocyanin production and differentiation of trichome cells	[16]
C24	NA	PtrMYB097 and 101	MaMYB52(TTL2)	**–**	**–**
C25(S5)	AtMYB123	PtrMYB086, 087 and 134	MaMYB50, 81, 82 and 99(TT2L1)	anthocyanin biosynthesis	[9, 24]
C26	NA	PtrMYB011, 129, 159, 171 and 178 and 183	MaMYB51 and 66	–	–
C27(S6)	AtMYB75, 90, 113 and 114	PtrMYB116, 117, 118, 119 and 120	MaMYB58(MYBA)	anthocyanin biosynthesis in vegetative tissues	[60, 61]
C28	AtMYB46 and 83	PtrMYB002, 003, 020 and 021	MaMYB72 and 85	triggers the expression of secondary cell wall related MYBs	[62]
C29	AtMYB26, 67 and 103	PtrMYB008, 010, 040, 103, 108, 127, 128 and 137	MaMYB26, 29, 30, 47 and 49	stamen endothecium lignification and tapetum and trichome development	[63–65]
C30	AtMYB18, 19 and 45	PtrMYB145	MaMYB21	response to infection with *Pseudomonas syringae*.	[18]
C31(S13)	AtMYB50, 55, 61 and 86	PtrMYB042, 055, 074, 121, 148, 170 and 216	MaMYB67 and 87	stomatal aperture	[66, 67]
C32(S20)	AtMYB2, 62, 78, 108, 112 and 116	PtrMYB034, 036, 066, 142, 164, 202 and 210	MaMYB40, 41, 74 and 95	response to fungal attack	[61, 68, 69]
C33(S19)	AtMYB21, 24 and 57	PtrMYB204 and 207	MaMYB8	flower-specific transcription factor, is regulated by COP1	[70, 71]
C34(S18)	AtMYB33, 65, 81, 97, 101, 104 and 120	PtrMYB007, 012, 024, 110, 124 and PtrMYB3R03	MaMYB2, 17 and 25	anther and pollen development. Response to ABA, anoxia and cold stress	[1]
C35	AtMYB71 and 79	PtrMYB014, 015,098, 195, 214 and 229	MaMYB12	stress response	[72]
C36	AtMYB27	PtrMYB103	MaMYB45	Phenylpropanoid pathway	[73]
C37	AtMYB48, 59,	PtrMYB023 and 206	MaMYB69	Stress response	[74–76]
C38	AtMYB125	PtrMYB208 and 218	MaMYB84	male gametophyte development	[17]
C39(S25)	AtMYB22, 98, 100, 115 and 118	PtrMYB001, 004, 022, 073, 147 and 151	MaMYB7, 68 and 88	Induction of green root and petal development	[8]
C40(S22)	AtMYB44, 70, 73 and 77	PtrMYB019, 029, 033, 105, 122. 140, 173, 176 and 177	MaMYB23, 33,56, 57,62, 78, 79 and 80	stress responses, and leaf senescenceis	[77–79]
C41(S23)	AtMYB1, 25 and 109	PtrMYB041 and 163	MaMYB24 and 27	stress resistance-related transcription.	
C42(S21)	AtMYB52, 54, 56 69, 105, 110 and 117	PtrMYB026, 031, 039, 052, 062, 082, 090, 136, 158, 161, 167, 175 and 189	MaMYB35, 55, 96, 102 and 103	lignin, xylan and cellulose biosynthesis,	[9, 80]
C43/44	AtMYB91	PtrMYB091, 154 and 221	MaMYB13	cell differentiation	[8]
C45	AtMYB88, 89 and 124	PtrMYB196 and 213	MaMYB37	cell proliferation in the stomatal lineage and cold hardiness	[8, 81]
S12	AtMYB28, 29, 34, 51,76 and 122	NA	MaMYB91	Seed development and aliphatic glucosinolate biosynthesis	[82]
Ma-1	NA	NA	MaMYB65 and 101	–	–
Ma-2	NA	NA	MaMYB53 and 54(TT2L3)	–	–
3R-related	AtMYB3R1, 2,3, 4 and 5	PtrMYB3R01, 02, 04 and 05 and PtrMYB131, 231 and 232	MaMYB3R1, 2,3 and 4	coordinate cell proliferation with differentiation in shoot and root	[83]

### Promoter analysis and gene structures of *R2R3* and *3R-MYB*s

**Fig. 2:**
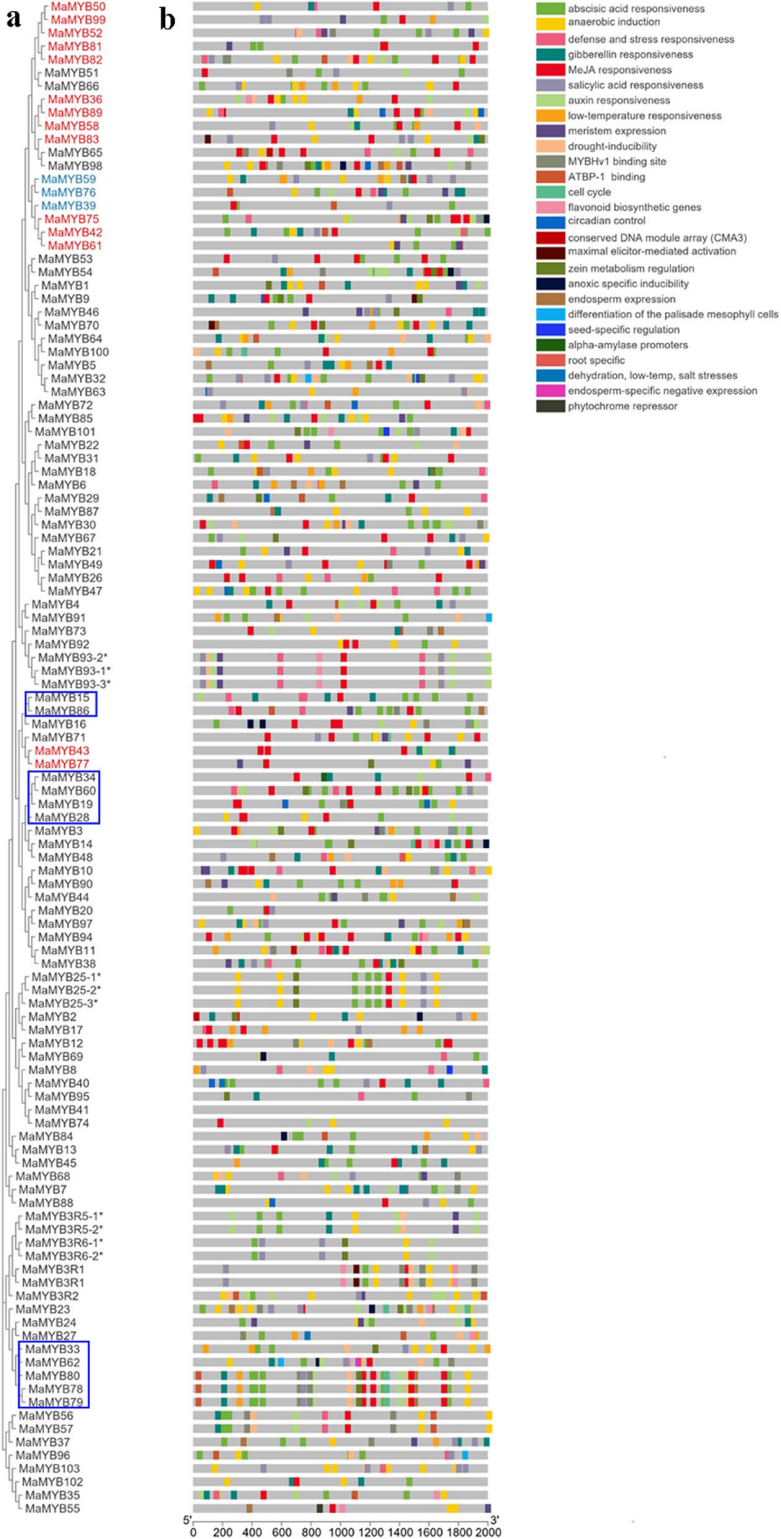
Phylogenetic relationships and cis-elements in promoter regions of R2R3 and 3R-MaMYBs. **A** Phylogenetic tree using 107 R2R3 and 3R-MaMYBs. **B** Cis-element distribution in the promoter regions of *R2R3* and *3R-MaMYB*s

**Fig. 3:**
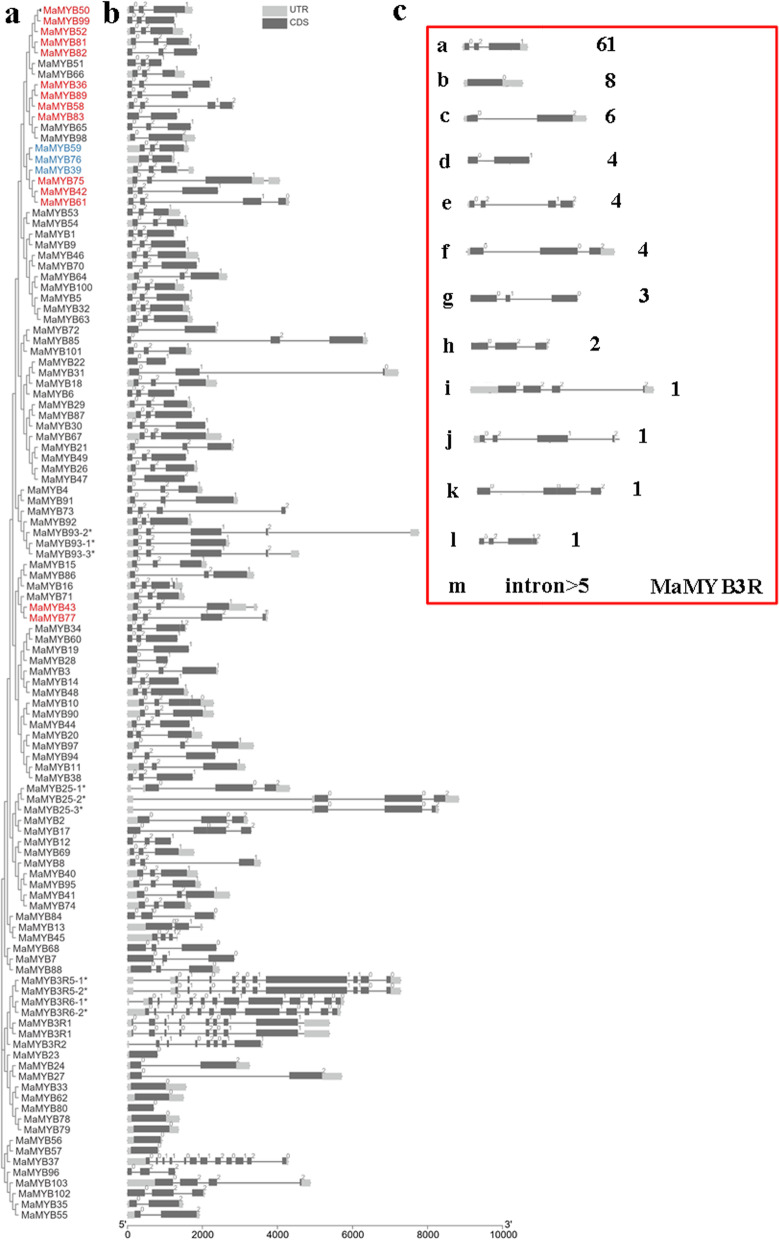
Phylogenetic relationships and gene organization of R2R3 and 3R-MaMYBs. **A** Phylogenetic tree using 107 R2R3 and 3R-MaMYBs. **B** Exon/intron structures of *Populus R2R3-MYB*s. **C** Schematic of intron distribution patterns of *R2R3* and *3R-MaMYB*s

Promoter regions (2000 bp upstream) of *R2R3* and *3R-MaMYB*s were extracted and the cis-elements were predicted. Light-response related cis-elements made up the greatest proportion (48%) of detected cis-elements (Additional file 4). Thirty-one classes of cis-elements were identified and the hormone related cis-elements were widely detected in the promoter regions of *MaMYB*s (Fig. 2). *R2R3-MaMYB*s with annotated functions of stress-responsiveness (blue boxes) had more abscisic acid (ABA), salicylic (SA) acid or methyl jasmonate (MeJA) related cis-elements. In particular, *MaMYB78*, *79,* and *80*, which were annotated as *MYB*s involved in stress responses and leaf senescence, had responsive cis-elements for all ABA, SA, MeJA, auxin, and gibberellin. *MaMYB*s with putative functions of regulating phenylpropanoid pathway were indicated by reddish shadows and blue shadows (for repressors). These *R2R3*-*MaMYB*s also possessed MeJA, ABA and stresses related cis-elements. The intron/exon organizations of *R2R3* and *3R-MaMYB*s were detected, and the exon phases were also indicated (Fig. 3). Thirteen intron/exon patterns (a–m) were summarized, among which, an “a” pattern (two introns with phase numbers of 0 and 2) was the prevalent pattern for *MaMYB*s at 63.5%. All *3R*-*MaMYBs* belonged to “m” pattern and possessed more than five introns.

### Chromosomal distribution and synteny analyses of *R2R3* and *3R*-*MaMYB*s

**Fig. 4:**
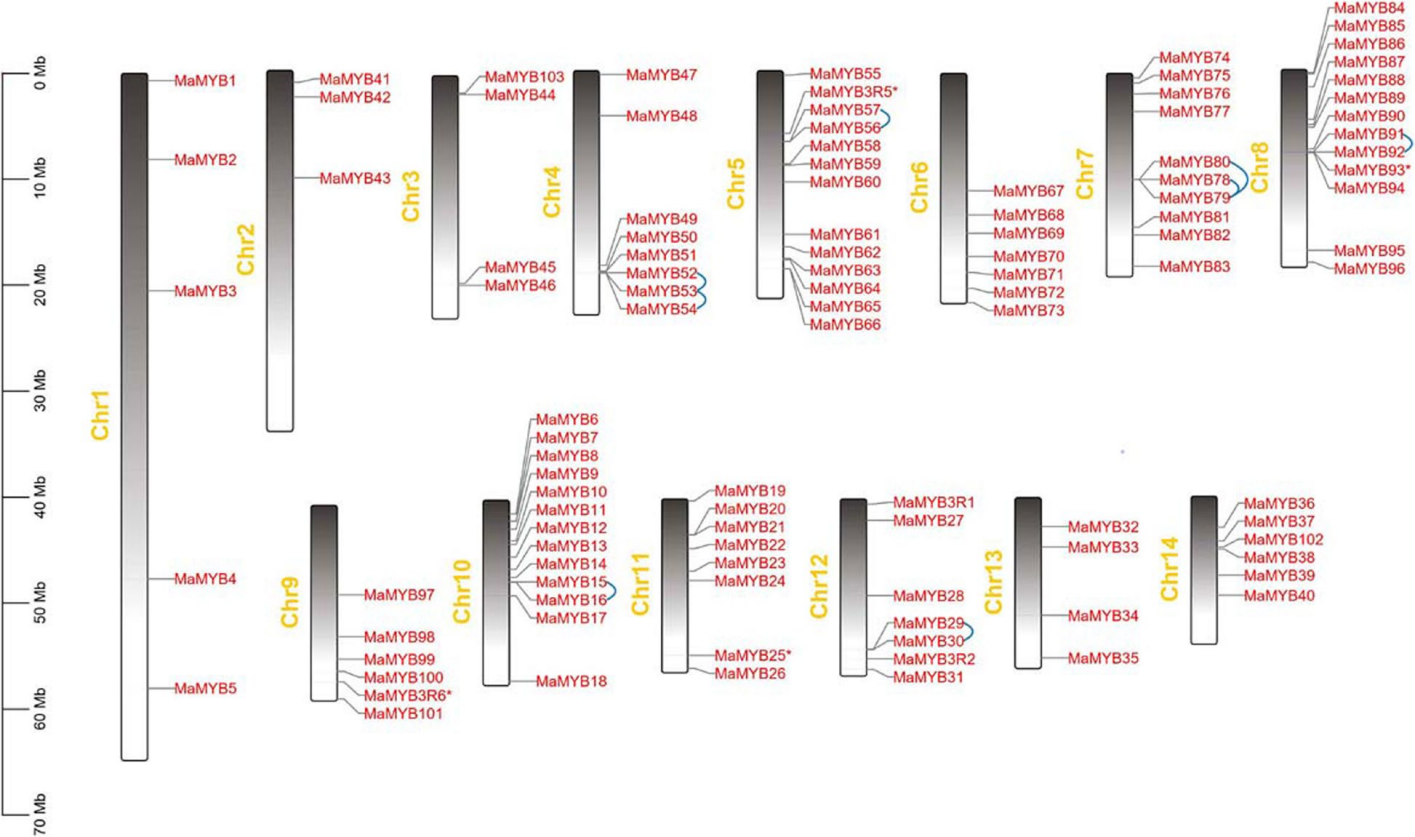
Distribution of *R2R3* and *3R-MaMYB*s in *Morus alba* chromosomes. The tandem gene pairs are linked by blue lines

**Fig. 5:**
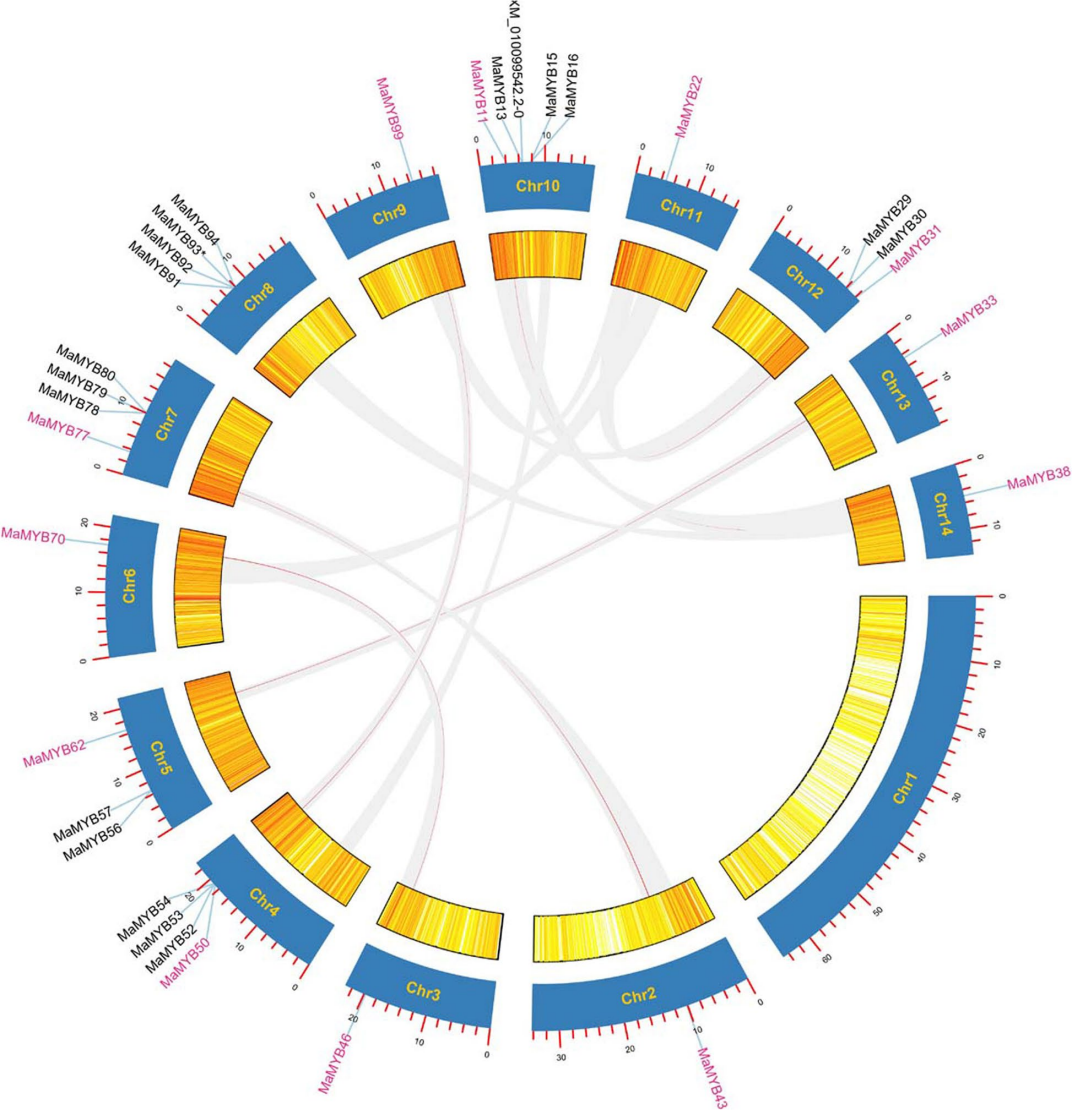
Schematic representations of collinear relationships of *R2R3* and *3R-MaMYB*s. Gray and red lines represent all synteny blocks and duplicated *R2R2-MYB* gene pairs in the *Morus* genome, respectively. The gene density is indicated in the heatmap by the inner circle. The corresponding chromosome number is shown on each chromosome

All 107 *R2R3* and *3R-MaMYB*s were mapped to 14 chromosomes based on the genome information. Each chromosome contained several *MaMYB*s with nonuniform distribution (Fig. 4). Chromosomes 1 and 2, the top two longest chromosomes occupying 30.37% of the whole genome, contain only five and three *MaMYB*s, respectively (7.17% of total *R2R3* and *3R-MaMYB*s). Chromosomes 5, 8, and 10 held 13, 14 and 14 members each, respectively, and had the densest distribution of *MaMYB*s. This nonuniform distribution of *MYB*s was also observed in other plants including *Z. may* and *Populus* [10, 12]. Whole genome duplication (WGD) is the main force driving gene family duplication and is important for neo-function gene occurrence. The duplication events for *MaMYB*s were detected. Duplicated gene pairs resulting from intra-chromosome duplication and inter-chromosome block duplication were revealed (Additional file 5). Six tandem events in 16 *MaMYB*s and six block duplication gene pairs in 12 *MaMYB*s were found for *R2R3-MaMYB*s (Figs. 4 and 5). Compared with 145 segmental duplication events with 156 *R2R3-MYB* in *Populus*, fewer duplication events for *MaMYB*s occurred because the *M. alba* lineage underwent no WGDs after its separation from the Eurosid I clade, while *Populus* underwent a recent WGD event [25, 84].

### Expression profiles of *R2R3* and *3R-MaMYB*s

**Fig. 6:**
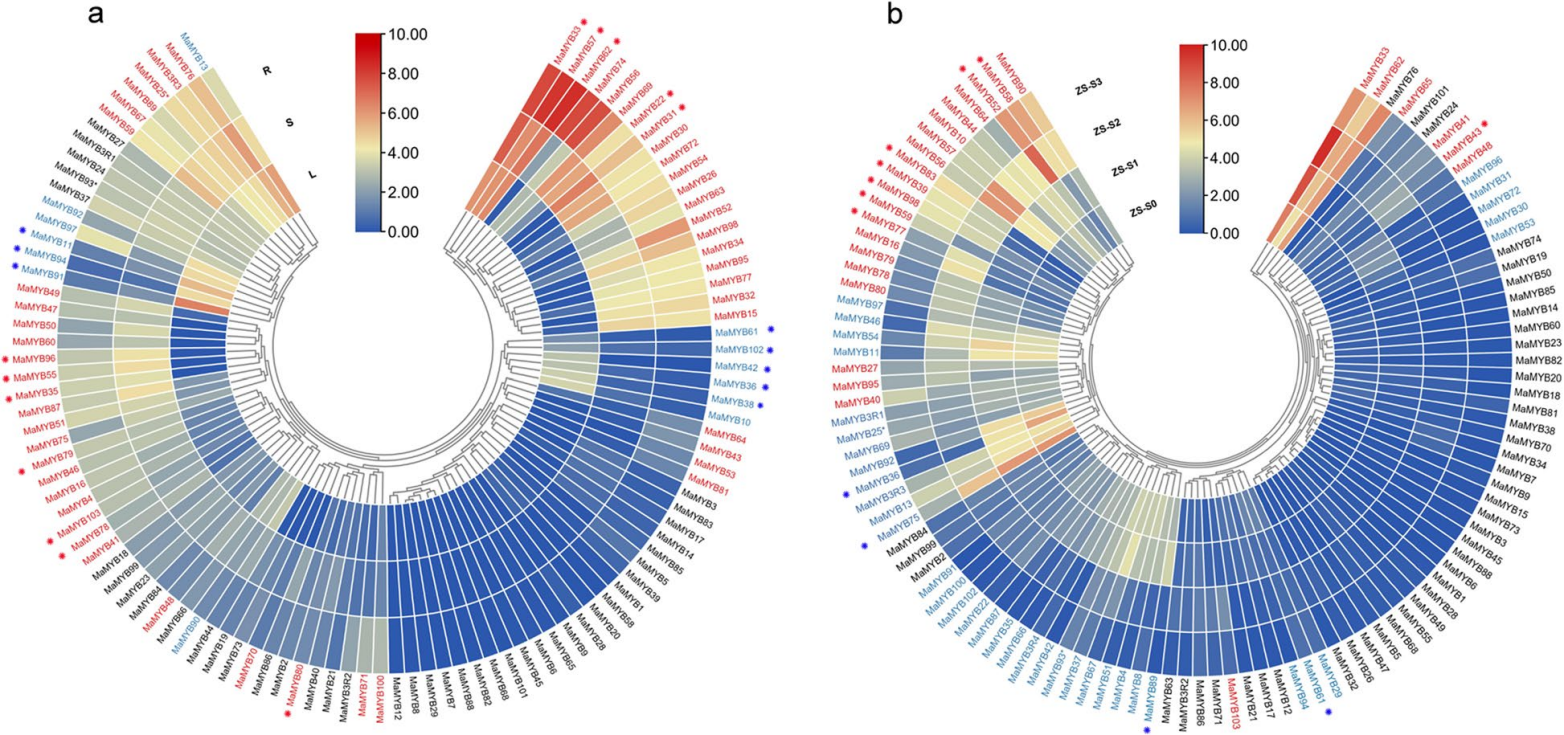
Expression profiles of *R2R3* and *3R-MaMYB*s in *Morus*. **A** Heatmap of *R2R3* and *3R-MaMYB* expression levels in *Morus* roots, stems, and leaves. *MaMYB*s with higher expression levels in leaves are shown in blue, and *MaMYB*s with higher expression levels in roots or stems are shown in red. *MaMYB*s referred to in the text are also indicated by stars. **B** Heatmap of *R2R3* and *3R-MaMYB* expression levels in fruits at different developmental stages. The *MaMYB*s with higher expression levels in S0 or S1 are colored with blue, and the *MaMYB*s with higher expression levels in S2 or S3 are colored with red. Differentially expressed *MaMYB*s involved in the phenylpropanoid pathway are indicated by stars

A spatial expression profile based on RNA-seq data of roots, stems and leaves showed that 66 of 107 *R2R3* and 3*R-MaMYB*s exhibited organ preference in expression. Among these organ-preferential differentially expressed genes, only 14 *MaMYB*s (marked with blue colors) showed higher expression levels in leaves (Fig. 6A and Additional file 6). These *MaMYB*s may be responsible for specific metabolite accumulation in leaves. For example, *MaMYB11*, *38* and *94* (C2/S9) were annotated as *MYB*s involved in cuticular wax biosynthesis and showed significant preference in leaves. Flavonoid biosynthesis related *MYB*s, including *MaMYB36*, *42*, *61,* and *102* had the highest expression levels in leaves rather than in stems and roots. *MaMYB91*, which was annotated as an aliphatic glucosinolate biosynthesis related *MYB,* was also preferentially expressed in leaves. Most *R2R3*-*MaMYB*s with higher expression levels in roots or stems were *MYB*s involved in secondary cell wall components and stress responsiveness. For example, cell wall component biosynthesis related *MYB*s, including *MaMYB35*, *55,* and *103* (C42/S21) as well as *MaMYB22* and *31* (C7) and stress responsiveness related *MYB*s, including *MaMYB33*, *35*, *57, 62*, *78*, *79,* and *80* (C40/S22) and *MaMYB4* (C22), showed higher expression levels in roots or stems (Fig. 6 A). It was obvious that *R2R3-MaMYB*s with similar annotated functions always maintained similar expression patterns in different organs.

**Fig. 7:**
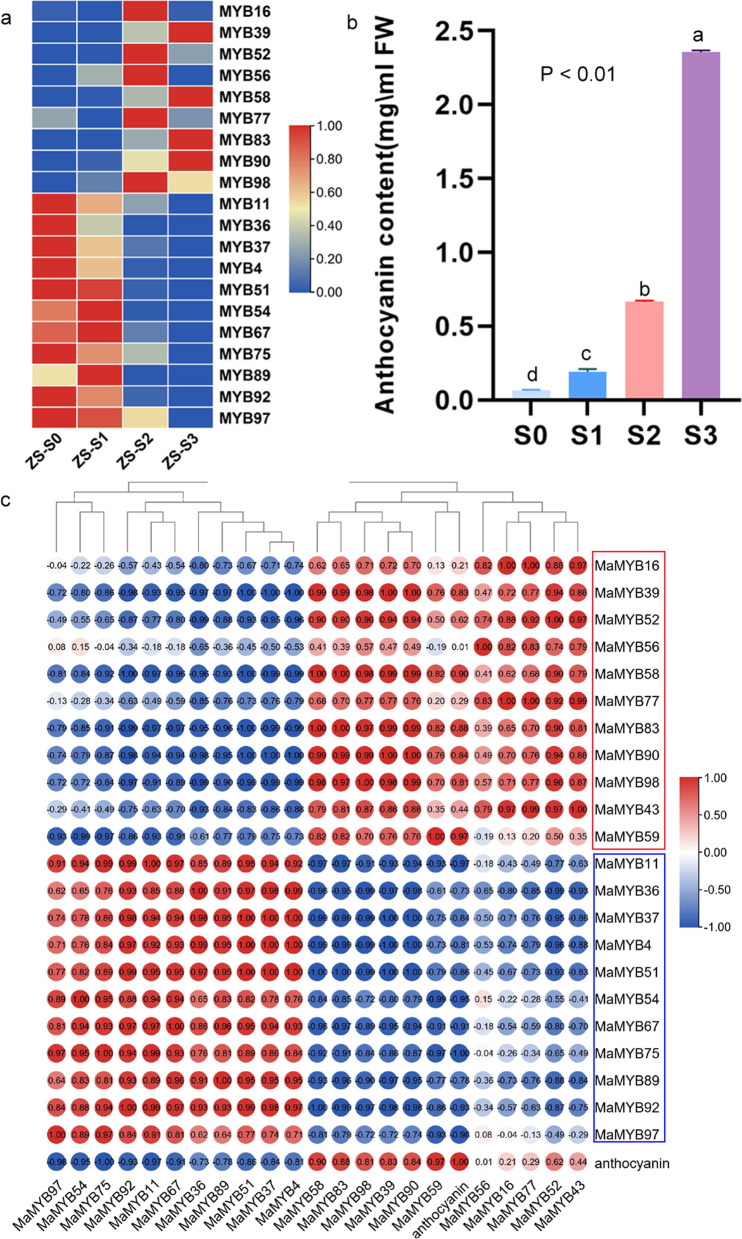
Relationships between the expression levels of these *MaMYB*s and anthocyanin accumulation during mulberry fruit ripening. **A** Expression levels of selected *MaMYB*s during fruit ripening using qRT-PCR. **B** Changes in anthocyanin contents of mulberry fruits during fruit ripening. **C** Correlation between the expression levels of selected *MaMYBs* and anthocyanin accumulation. The pearson correlation coefficients were marked

Mulberry fruits are rich in flavonoids and anthocyanins and are used for wine, nutrient food, and medicine. The expression patterns of *R2R3-MaMYB*s and *3R-MaMYB*s were revealed, and 72 of 107 *R2R3* and *3R-MaMYB*s were identified as DEGs during mulberry ripening (Fig. 6B and Additional file 6). Two clusters of *R2R3* and *3R-MaMYB*s based on the expression patterns, 27 early-expression *MaMYBs*, *MaMYB*s with higher expression levels in S0 or S1 and 45 late-expression *MaMYB*s, *MaMYB*s with higher expression levels in S2 or S3, were marked using red and blue respectively. Twenty differentially expressed *R2R3-MaMYB*s involved in flavonoid biosynthesis, anthocyanin biosynthesis, fruit development, and stress responsiveness and with relatively high overall expression levels were selected for qRT-PCR validation (Fig. 6B, Fig. 7A and Additional files 7 and 8). The mulberry fruit ripening process occurs along with the accumulation of anthocyanin and color changes (Fig. 7B). Both RNA-seq and qRT-PCR showed that *R2R3-MaMYB*s with putative roles in the phenylpropanoid pathway had different expression patterns during fruit ripening. The correlation relationships of the expression levels of these *MaMYBs* and anthocyanin contents were also revealed (Fig. 7C). It was clear that *MaMYB*s with late-expression patterns were positively correlated with anthocyanin accumulation, while *MaMYB*s with early-expression pattern are negatively correlated with anthocyanin accumulation (Fig. 7C). *R2R3-MaMYB*s such as *MaMYB43*, *77*, *83*, *98,* and *58,* which may play direct roles in PA or anthocyanin accumulation, showed late-expression patterns. These *MaMYB*s are likely to positively regulate anthocyanin biosynthesis. For instance, MaMYB58 (MYBA) has been reported to interact with bHLH3 to activate the expression of anthocyanin biosynthetic genes to control anthocyanin biosynthesis. Moreover, some phenylpropanoid pathway repressors, such as *MaMYB59* and *MaMYB39,* also showed increasing expression levels along with fruit ripening. *MaMYB59* was annotated as a repressor of lignin biosynthesis, and *MaMYB39* was annotated as a repressor of flavonoids biosynthesis. In addition, there were also early-expression pattern *R2R3-MaMYB*s involved in flavonoid biosynthesis or anthocyanin biosynthesis. *MaMYB75* (*MYBF*), which was reported to be responsible for flavonol accumulation and negatively regulate anthocyanin biosynthesis, showed a decreasing expression level along the fruit ripening. *MaMYB36* and *89* were annotated as having functions in anthocyanin biosynthesis and trichome cell differentiation, and also exhibited early-expression patterns, indicating that *MaMYB36* and *8*9 may have been responsible for mulberry fruit trichome development rather than anthocyanin biosynthesis. The different expression patterns of flavonoid related R*2R3-MaMYB*s along with mulberry fruit ripening suggested that different *R2R3-MaMYB*s coordinated to maintain homeostasis and avoid the overaccumulation of anthocyanins during fruit ripening.

## Discussion

Genome-wide identification of R2R3-MYB TFs is influenced by quality of genome annotation and assembly [5]. A previous study identified 116 R2R3-MYBs based on de novo transcriptome data [23]. However, in the present study, we identified 103 R2R3-MYBs in *M. alba* using the latest *M. alba* genome assembly with a stricter workflow. Sequence length and MEME motif extraction validation were considered as additional two filtering steps to further screen MYBs in this study. The numbers of different kinds of MYBs are close in different plant species, except for *Populus* (Table 1). Moreover, the number of R2R3-MYBs (103) identified in *Morus* was close to that of *V. vinifera* (108), but far less than that of *Populus* (198). Although the number of R2R3-MaMYBs in *Morus* was less than that of *Populus*, the R2R3-MaMYBs covered almost all subgroups of *Populu*s R2R3-MYBs and *Arabidopsis* MYBs (Table 2). It has been suggested that WGDs and tandem duplication events have contributed to the expansion of R2R3-MYBs in land plants [85]. It was reported that no lineage-specific WGD occurred in *M. alba* after the γhexaploidization event [25]. Compared with duplication events identified for *R2R3-MYB*s in *Populus* which experienced a recent lineage-specific WGD [84], fewer duplication events were found in *Morus* for the *R2R3-MYB*s and only 28 *MaMYB*s resulting from tandem duplication and block duplication were found for the *R2R3-MaMYB*s (Figs. 4 and 5). The above differences further support the hypothesis that the expansion of R2R3-MYB TFs in land plants has been subfamily/clade asymmetric and lineage specific [5].

The conserved DBD domain in the N-terminal of MYBs was identified in R2R3-MaMYBs. The spaced W forming WX_19_WX_19_W was highly conserved in R2 repeat, while the replacement of the first W with F, I, or M at position 54 for R3 was generally reported in plants [10–13]. The conserved spaced W motifs in R2 and R3 are signature sequences for the DBD domain of MYBs. In addition to the conserved W, there are several highly conserved residues in the DBD domain. These conserved residues mainly contained residues located at the start and end of helices and residues consisting of the third helix (Fig. 1). Residues located at the start and end of helices may be responsible for maintaining the helix structure. The conserved third helices in R2 and R3 are responsible for the specific base recognition in the major groove of the DNA [6]. In addition, the residues consisting of the third helix in R2 and those consisting of the third helix in R3 are greatly different which might be important for recognition of diverse DNA sequences. It has been reported that the residues including L39, N86, K89 and 90 in the third helices of R2 and R3 are important for maintaining MYB functions [7]. In addition, K35 was reported to play an important role in sensing DNA methylation at the fifth position of cytosine (5 mC) [7]. The InterPro MYB domain signature pattern (PS00037) was detected and conserved in the first helices of both R2 and R3 repeats. Given that the first helices were beyond the DNA binding regions, it was possible that the first helix functioned as structure helices.

A total of 103 *R2R3-MaMYB*s, except for *MaMYB53*, *54*, *65,* and *101,* were grouped with the R2R3-MYBs from *Arabidopsis* and *Populus,* indicating the high retention of *R2R3-MYBs* in *M. alba* afterγpaleohexaploidy [25]. *MaMYB53*, *54*, *65,* and *101* formed two *Morus* specific subgroups (Ma1–2). Because *R2R3-MYB* gene retention after duplication events is biased among angiosperm taxa, and the pervasive duplications of *R2R3-MYB*s in core eudicots are thought to be gamma-triplication derived [5], *R2R3-MYB*s in *Morus* seem to exhibit relatively conserved R2R3-MYBs from before the divergence of the core eudicots. Most *R2R3-MYB*s in *Arabidopsis* have been well annotated and functionally characterized [1, 9, 14]. Phylogenetic analysis based functional annotation suggested that R2R3-MaMYBs were mainly involved in responses to abiotic and biotic stresses, development and secondary metabolite biosynthesis (Table 2). *AtMYB20, 41* and *44* were reported to involve in response to abiotic stresses and *AtMYB44* was reported to enhance stomatal closure to confer abiotic stress tolerance [38, 45, 86, 87]. Wheat *TaMYB31* was reported to involve in drought stress responses [81] *MaMYB14, 48*, *79* and *80* were annotated as MYBs involved in abiotic stress responses in mulberry. *AtMYB30* was reported to act as positive regulator of hypersensitive cell death program in plants in response to pathogen attack [50]. *MdMYB30* in apple and *SlMYB28* in tomato regulated plant resistance to pathogen attack [88, 89]. *MaMYB10, 44* and *90* as homologs clustered with *AtMYB30* may also function in response to pathogen attack. Mulberry is rich in phenylpropanoid-derived bioactive compounds, such as flavonoids and polyphenols, and mulberry fruits are known for their abundance of anthocyanins [90, 91]. Possible anthocyanin homeostasis was maintained by both cooperative and antagonistic interactions of phenylpropanoid pathway related R2R3-MaMYBs. Both the increasing expression of activators such as *MaMYB43*, *77*, *83*, *98* and *58* and decreasing expression levels of *MaMYB54* (*TT2L3*), which was involved in flavonol biosynthesis along with fruit ripening, positively affected anthocyanin accumulation. In contrast, increasing the expression levels of repressors such as *MaMYB39* (*MYB4*) and *MaMYB59* helped to avoid the overaccumulation of anthocyanins and preserve the balance between lignin biosynthesis and flavonoid biosynthesis (Fig. 6). Both our expression profiles and a previous study on *MaMYB*54 (*TT2L3*) and *MaMYB39* (*MYB4*) support this postulation [24]. The homeostasis between anthocyanin and lignin biosynthesis regulated by cooperation of repressor *MdMYB16* and activator *MdMYB1* was also reported in apples [92].

## Conclusion

A comprehensive analysis of the *R2R3-MYB* gene family using the latest *M. alba* chromosome-level genome was conducted in the present study. In total, 166 *MYB* genes were identified and 103 *R2R3-MaMYB*s and four *3R-MaMYB*s were further analyzed. Functional annotation for *R2R3-MaMYB*s based on phylogenetic analysis as well as functional annotation of orthologs from *Arabidopsis* and *Populus* suggested the possible functional subgroups of R2R3-MaMYBs. Conserved gene organization, promoter analysis, and expression profiles also provided evidence for putative functions. Moreover, *R2R3-MYBs* involved in the phenylpropanoid pathway were investigated, and MaMYBs involving in flavonoid biosynthesis and anthocyanins accumulation were identified based on functional annotation and the correlation of their expression levels with anthocyanin contents. These findings will be valuable for future genetic improvement of specific biological processes, such as flavonoid or anthocyanin biosynthesis, and provide a basic reference for research on the functional characterization of *MaMYB* genes involved in specific biological process.

### Ethical statement for experimental research and field studies on plants

Experimental research and field studies on plants complies with relevant institutional, national, and international guidelines and legislation.

## Supplementary Information

**Additional file 1: Table S1.** Primers used in this study.

**Additional file 2: Table S2.** *MYB*s in *Morus alba*.

**Additional file 3: Fig. S1.** Phylogenetic tree of R2R3-MYBs from *Arabidopsis*, *Populus*, and *Morus*. The subgroups are indicated based on both the classification in *Arabidopsis* and *Populus*.

**Additional file 4: Table S3.** Predicted cis-elements in promoter regions of *MaMYB*s.

**Additional file 5: Table S4.** Gene duplication in *Morus alba*.

**Additional file 6: Table S5.** Expression matrix of *MaMYB*s based on RNA-seq.

**Additional file 7: Fig. S2.** qRT-PCR results for 20 differentially expressed *R2R3-MaMYB*s during fruit ripening. The significance was indicated by different letters(*p* < 0.01).

**Additional file 8: Table S6.** Relative expression matrix for 20 differentially expressed *R2R3-MaMYB*s during fruit ripening.

## Data Availability

The datasets generated during the current study are available in the national genomics data center (NGDC) with accession number: CRA006074 (https://ngdc.cncb.ac.cn/ search/?dbId = gsa&q = CRA006074).
