# Supplementary figures and images for: Comprehensive analysis of the MYB transcription factor gene family in Morus alba
Source: BMC Plant Biol. 2022 Jun 8;22:281. doi: 10.1186/s12870-022-03626-5 (PMC9175366; doi:10.1186/s12870-022-03626-5)

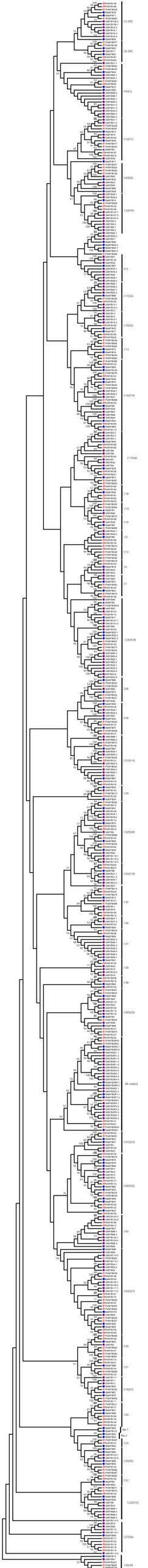

Supplement: Supplementary file 3 — Additional file 3: Fig. S1. Phylogenetic tree of R2R3-MYBs from Arabidopsis, Populus, and Morus. The subgroups are indicated based on both the classification in Arabidopsis and Populus. [file 12870_2022_3626_MOESM3_ESM.pdf]

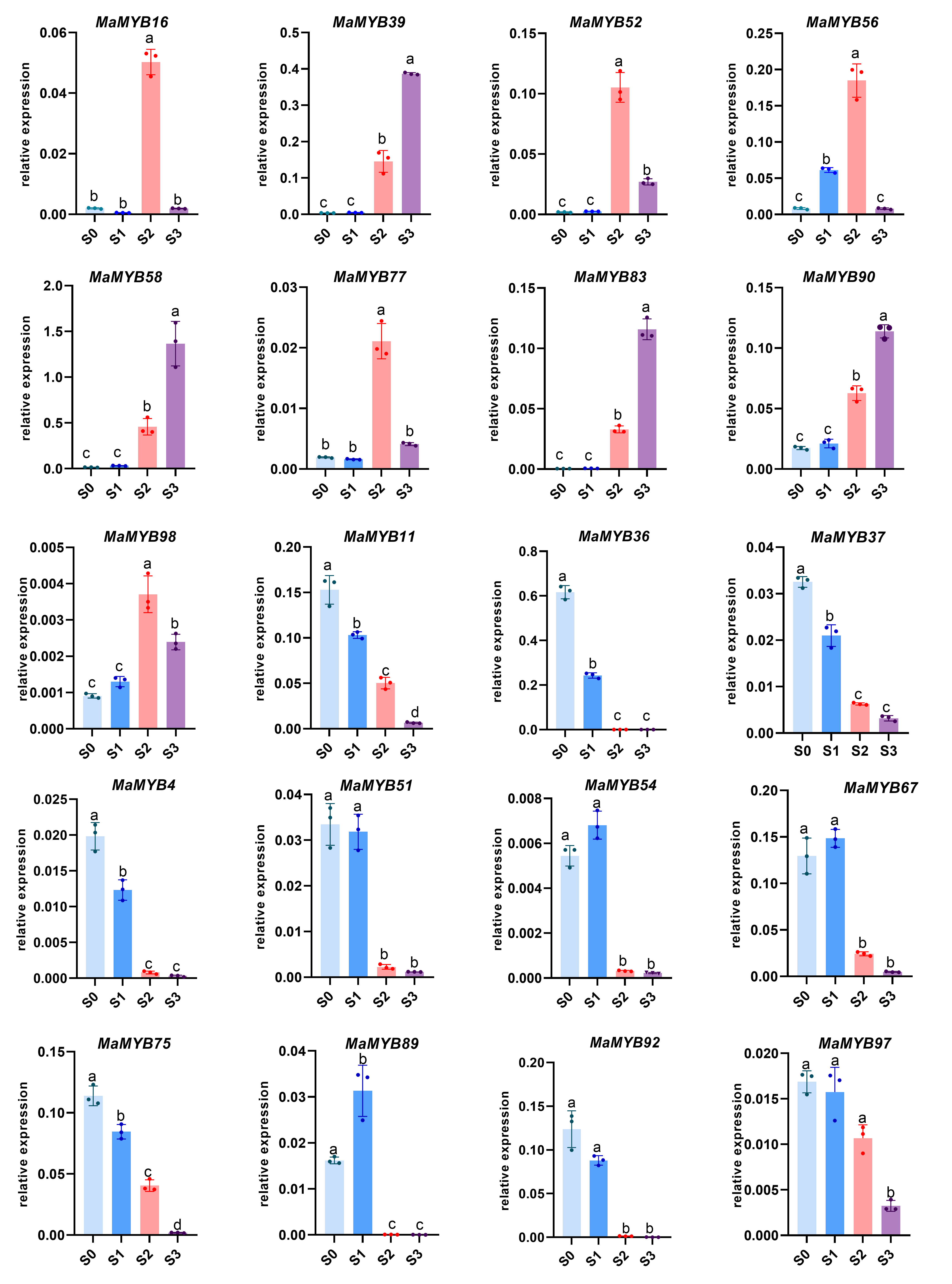

Supplement: Supplementary file 7 — Additional file 7: Fig. S2. qRT-PCR results for 20 differentially expressed R2R3-MaMYBs during fruit ripening. The significance was indicated by different letters(p < 0.01). [file 12870_2022_3626_MOESM7_ESM.tif]
